# Lyso-GM2 Ganglioside: A Possible Biomarker of Tay-Sachs Disease and Sandhoff Disease

**DOI:** 10.1371/journal.pone.0029074

**Published:** 2011-12-20

**Authors:** Takashi Kodama, Tadayasu Togawa, Takahiro Tsukimura, Ikuo Kawashima, Kazuhiko Matsuoka, Keisuke Kitakaze, Daisuke Tsuji, Kohji Itoh, Yo-ichi Ishida, Minoru Suzuki, Toshihiro Suzuki, Hitoshi Sakuraba

**Affiliations:** 1 Department of Analytical Biochemistry, Meiji Pharmaceutical University, Tokyo, Japan; 2 Department of Clinical Genetics, Meiji Pharmaceutical University, Tokyo, Japan; 3 Department of Molecular Medical Research, Tokyo Metropolitan Institute of Medical Science, Tokyo, Japan; 4 Department of Medicinal Biotechnology, Institute for Medicinal Research, Graduate School of Pharmaceutical Sciences, The University of Tokushima, Tokushima, Japan; 5 Department of Molecular Biochemistry, Meiji Pharmaceutical University, Tokyo, Japan; 6 Disease Glycomics Team, Systems Glycobiology Research Group, RIKEN, Saitama, Japan; Baylor Research Institute, United States of America

## Abstract

To find a new biomarker of Tay-Sachs disease and Sandhoff disease. The lyso-GM2 ganglioside (lyso-GM2) levels in the brain and plasma in Sandhoff mice were measured by means of high performance liquid chromatography and the effect of a modified hexosaminidase (Hex) B exhibiting Hex A-like activity was examined. Then, the lyso-GM2 concentrations in human plasma samples were determined. The lyso-GM2 levels in the brain and plasma in Sandhoff mice were apparently increased compared with those in wild-type mice, and they decreased on intracerebroventricular administration of the modified Hex B. The lyso-GM2 levels in plasma of patients with Tay-Sachs disease and Sandhoff disease were increased, and the increase in lyso-GM2 was associated with a decrease in Hex A activity. Lyso-GM2 is expected to be a potential biomarker of Tay-Sachs disease and Sandhoff disease.

## Introduction

Hydrolysis of GM2 ganglioside (GM2) is catalyzed in lysosomes by β-hexosaminidase (Hex, EC 3.2.1.52) A, and a non-enzymic factor, GM2 activator (GM2A), is essentially required in the catabolic reaction *in vivo*. Hex A is a heterodimeric protein composed of an α- and a β-subunit encoded by the *HEXA* and *HEXB* genes, respectively, and GM2A is encoded by the *GM2A* gene. Defects in any of these three genes result in excessive accumulation of GM2 and related glycolipids in lysosomes, mainly those of neural cells, leading to a rare neurodegenerative disorder, GM2 gangliosidosis, which comprises three biochemically different disorders: (a) Tay-Sachs disease (B variant, MIM 272800) results from mutations of the *HEXA* gene, being associated with deficient Hex A activity; (b) Sandhoff disease (0 variant, MIM 268800) is due to mutations of the *HEXB* gene, being associated with deficient Hex A and Hex B (a homodimer of the β-subunit) activities; and (c) GM2A deficiency (AB variant, MIM 272750) is an extremely rare genetic disease caused by mutations of the *GM2A* gene, being characterized by lysosomal storage of GM2 despite normal Hex A activity. GM2 gangliosidosis exhibits a wide clinical spectrum from the early-onset severe “infantile type” to the late-onset mild “attenuated type”. The clinical, genetic, and biochemical aspects of this disease have been reviewed [1].

So far, little effective therapy for GM2 gangliosidosis has been developed [2]–[5]. However, recently the therapeutic potential of enzyme replacement therapy (ERT) with recombinant human Hex A and modified Hex B, both of which exhibit GM2-degrading activity, has been reported [6]–[8], and they are promising as new enzymes for ERT for Tay-Sachs disease and Sandhoff disease. Considering this, a useful biomarker for the diagnosis of and monitoring of the response to therapy for these diseases is strongly required.

In this study, we paid attention to lyso-GM2 ganglioside (lyso-GM2), a derivative of GM2 that recently attracted interest as a possible pathogenetic agent for these diseases, and measured the lyso-GM2 levels in the brain and plasma in Sandhoff mice, and examined the effect of intracerebroventricularly administrated modified Hex B on the degradation of the lyso-GM2 accumulated in the brain and plasma. Then, we determined the levels of lyso-GM2 in plasma samples from patients with various types of GM2 gangliosidosis including Tay-Sachs disease, Sandhoff disease, and GM2A deficiency.

## Materials and Methods

### Ethics

The human blood samples were obtained before 1999 with verbal consents from the participants and/or parents of the infants and had been stored in frozen state until the study. The Ethical Committee of Meiji Pharmaceutical University discussed about the usage of these samples for measuring the concentration of lyso-GM2 and activity of hexosaminidase, and approved it (ID: 1908) because at present the written consent from the participants and/or parents of the infants cannot be obtained as they are dead and/or their present addresses are unknown but this study is thought to be useful for medicine and these samples are essentially required for this study.

This study involving animals was approved by the Animal Experiment Committee of the University of Tokushima (ID: 10106), and the experiments were performed according to protocols approved by the committee.

### Materials

Lyso-GM2, as a standard for both high performance liquid chromatography (HPLC) and Matrix-Assisted Laser Desorption/Ionization-Time of Flight-Mass Spectrometry (MALDI-TOF-MS), was purchased from TAKARA BIO Inc. (Shiga, Japan). GM2, as a standard, for thin layer chromatography, and 4-methylumbelliferyl-N-acetyl-β-D-glucosaminide (MUG), for determining Hex A and Hex B activities, were obtained from Sigma Chemical Co. (St. Louis, MO). 4-Methylumbelliferyl-6-sulfo-β-D-glucosaminide (MUGS), for measuring Hex A activity, was purchased from Calbiochem (San Diego, CA). *o*-Phthalaldehyde (OPA), for derivatizing lyso-GM2, was purchased from Nacalai Tesque (Kyoto, Japan). 2,5-Dihydroxybenzoic acid (2,5-DHB) and Peptide calibration standard II for MALDI-TOF-MS were purchased from Bruker Daltonics Inc. (Billerica, MA). All other chemicals used were of analytical grade. A genetically engineered *HEXB* encoding a chimeric human β-subunit containing a partial amino acid sequence of the α-subunit (βD452N, βL453R, and βRQNK312-315 GSEP) was designed by structure-based homology modeling, and was introduced into Chinese hamster ovary cells (RIKEN BIORESOURCE CENTER CELL BANK, Tokyo, Japan), and a modified Hex B was produced and purified as described previously [4].

### Human plasma samples

Plasma samples for measurement of the MUG-degrading and MUGS-degrading activities, and the lyso-GM2 levels were obtained from two patients with Sandhoff disease (one infantile [9] and one adult [10] case) and their parents, five patients with Tay-Sachs disease (five infantile cases) and their parents, one patient with GM2A deficiency (one infantile case) [11], and 48 control subjects.

### Measurement of lyso-GM2 in mouse brain and plasma

Sandhoff (*Hexb^−/−^*) mice (C57BL/6×129sv) were kindly provided by Dr. Richard L. Proia (NIDDK, National Institute of Health). Ten-week-old Sandhoff mice (n = 3) were intracerebroventricularly injected with a modified Hex B (20 µmol/h/25 µL), and then sacrificed 1 week after the injection, and their brains and plasma samples were obtained. Untreated Sandhoff mice (n = 5) and wild-type (C57BL/6) ones (n = 5) were also used in this experiment. Before removing their brains, the mice were perfused with phosphate-buffered saline (PBS), pH 7.4. Extraction of lyso-GM2 from the brain and plasma was performed according to our method for measurement of globotriaosylsphingosine [12], [13] with slight modifications. Briefly, to 10 µL of a brain homogenate, 120 µL of chloroform/methanol (1/2, v/v) was added, and then the mixture was centrifuged for 5 min at 1,000×g. The supernatant was placed in another tube. The residue was re-extracted with 120 µL of chloroform/methanol (2/1, v/v). To the combined supernatants, 120 µL of chloroform and 72 µL of water were added, and then the extract was centrifuged for 5 min at 2,000×g and the upper phase was removed. To the lower phase, 360 µL of methanol/water (2/1, v/v) was added, followed by mixing, and then the extract was centrifuged for 5 min at 9,000×g. Then, the combined upper phases were dried and taken up in 250 µL of water, and the water phase was extracted twice with 250 µL of water-saturated 1-butanol. The butanol phase was dried and dissolved in 50 µL of methanol, and then lyso-GM2 in the solution was derivatized with 25 µL of OPA reagent (pH 11). The OPA-derivatized lyso-GM2 was separated by HPLC, and then measured by means of fluorescence detection (excitation wavelength: 340 nm, and emission wavelength: 435 nm). Chromatographic separation was carried out on a Unison UK-C18 column (150×4.6 mm I.D., 3 µm; Imtakt, Kyoto, Japan) with a mobile phase of tetrahydrofuran/methanol/water (9/31/40, v/v/v, flow rate 1.0 mL/min). The column oven was kept at 40°C during the procedure.

For plasma lyso-GM2 extraction, 25 µL of plasma was used as a sample, and the extraction procedure was carried out in the same manner as in the case of brain lyso-GM2 except for the use of two volumes of the organic solvents. A calibration curve for lyso-GM2 was prepared by the addition of authentic lyso-GM2 to a normal brain homogenate or plasma.

### Identification of lyso-GM2 by MALDI-TOF-MS

To identify lyso-GM2, the crude lipid extract was applied to pre-coated high performance thin-layer chromatography (HPTLC)-silica gel plates (100×200 mm; E. Merck, Darmstadt, Germany). Then, lyso-GM2 was extracted twice from the HPTLC plates with 200 µL of water-saturated 1-butanol, and the butanol phase was dried. For both MS and MSMS measurements, dried samples were dissolved in 10 µL of the matrix solution (10 mg/mL 2,5-DHB in 50% methanol) and 4 µL of the resultant solution was loaded onto a sample plate with 384 sample positions, allowed to dry and then crystallized for 60 min at room temperature. Positive ion MALDI-TOF-MS and MS/MS spectra of the lyso-GM2 were obtained by ultrafleXtream (Bruker Daltonics Inc., Billerica, MA). In order to calibrate the mass spectra of the lyso-GM2, Peptide calibration standard II was used.

### Measurement of GM2 in mouse brain

Crude glycolipids were extracted from tissue homogenates according to the method previously described [11]. Briefly, crude glycolipids were extracted with a mixture of chloroform/methanol/water (4/8/3, v/v/v) three times. This solvent mixture containing the extracted crude glycolipids was collected and then dried under a stream of nitrogen. The crude glycolipids were resolved in chloroform/methanol/water (60/30/4.5, v/v/v), the concentration being 0.5 mg tissue weight/µL of the reagent. To identify GM2, the crude glycolipids were applied to precoated HPTLC-silica gel plates (100×200 mm; E. Merck). The developing solvent used was chloroform/methanol/water (65/25/4, v/v/v). Glycosphingolipids containing GM2 were visualized with orcinol reagent. Then, HPTLC-immunostaining with an anti-GM2 monoclonal antibody [14] was performed according to the method described previously [11], and the amounts of GM2 were determined by densitometric scanning with a luminescent image analyzer, LAS 4000, equipped with Multi Gauge Ver3.X (FUJI FILM, Tokyo, Japan).

### Measurement of lyso-GM2 in human plasma

The lyso-GM2 levels in human plasma samples were determined according to the method described under “Measurement of lyso-GM2 in mouse brain and plasma”.

### Enzyme assays

Total Hex (mainly Hex A and Hex B) and Hex A activities in human plasma samples were fluorometrically determined with MUG and MUGS as substrates, respectively, according to the method described previously [11].

### Statistical analyses

Statistical analyses were performed by means of Student's *t*-test. Values were considered statistically significant at P<0.05.

## Results

### Detection of lyso-GM2 in mouse brain

A lipid fraction including lyso-GM2 was obtained from brain tissues of mice and the free amine moiety of lysosphingolipids was labeled with OPA. Then, the lysosphingolipids were separated by HPLC, and the lyso-GM2 levels in the brain tissues from the Sandhoff mice treated and not treated with the modified Hex B and the wild-type ones were determined. Peak identification was performed by comparison with that of authentic lyso-GM2. The lyso-GM2 level was determined from the calibration curve obtained by plotting the lyso-GM2 peak area against the concentration.

**Figure 1:**
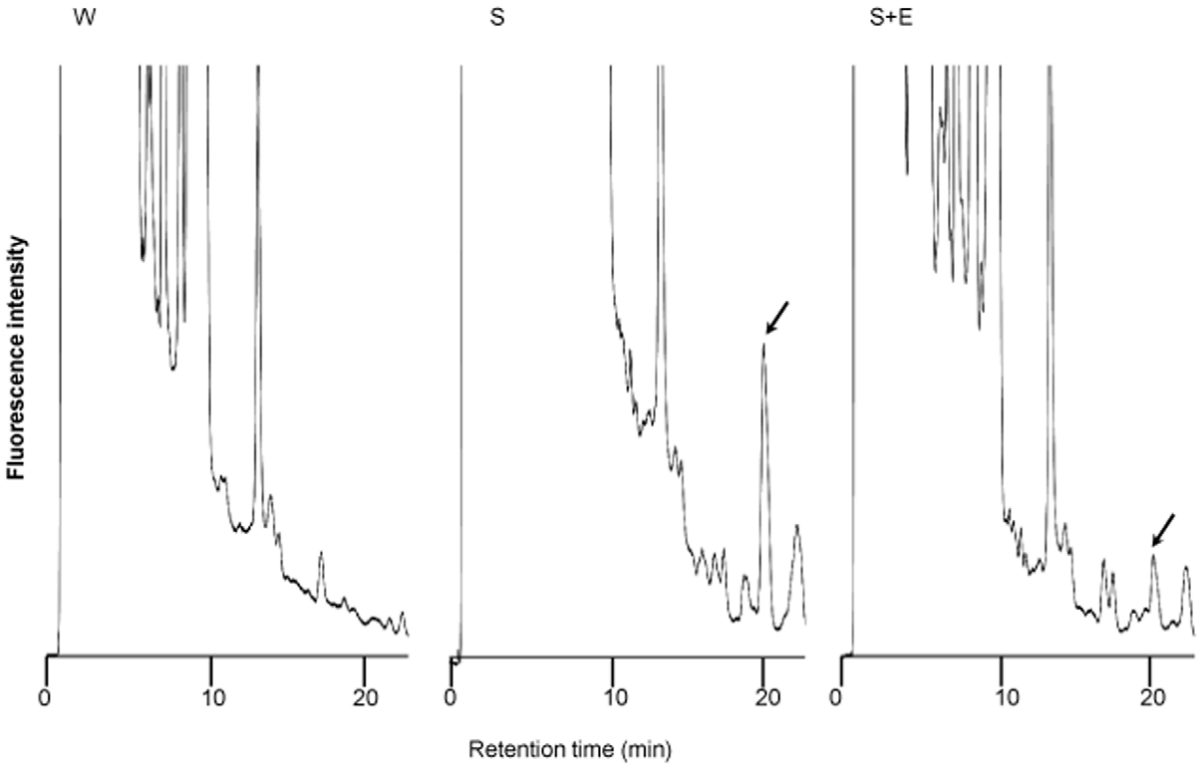
HPLC-chromatograms of lyso-GM2 in the brain. A wild-type mouse (W), an untreated Sandhoff mouse (S), and a Sandhoff mouse treated with the modified Hex B (S+E). Arrows indicate the peak of lyso-GM2.

Fig. 1 shows HPLC-chromatograms of lyso-GM2 in the brain tissues. A high peak was observed for the brain of the untreated Sandhoff mouse, the peak being apparently decreased for the brain of the enzyme-treated Sandhoff mouse. No peak corresponding to lyso-GM2 could be detected for the brain of the wild-type one.

**Figure 2:**
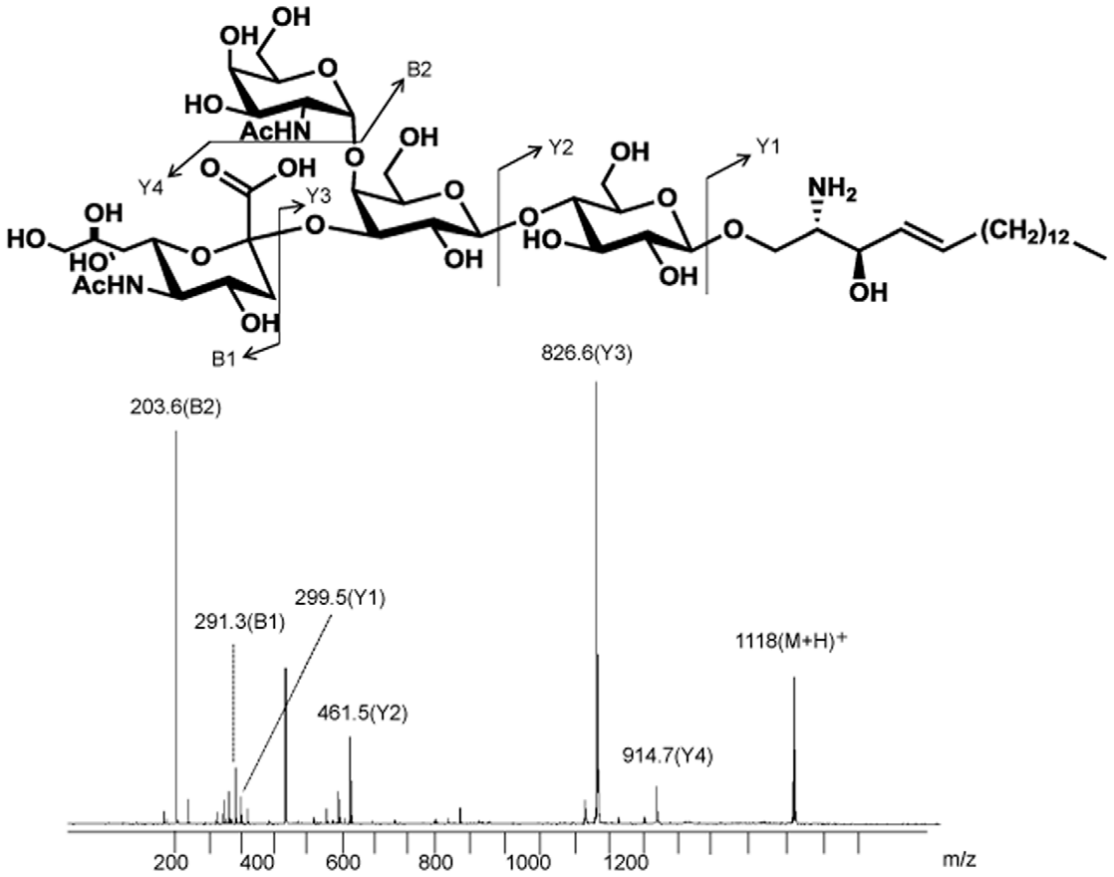
MALDI-TOF-MS spectra of lyso-GM2. Structure of lyso-GM2 (top) and its spectra obtained for the brain of a Sandhoff mouse (bottom).

MALDI-TOF-MS analysis was performed to confirm that the abnormal structure was lyso-GM2 accumulated in the brain tissues of Sandhoff mice. The spectrum exhibited a main [M+H]^+^ molecular ion signal at m/z 1118, and MALDI-TOF-MS analysis revealed that the sequence ions exhibited by the accumulated substrates in the Sandhoff mouse brain were identical to those derived from authentic lyso-GM2. The corresponding fragmentation scheme is shown in Fig. 2.

Lyso-GM2 levels in the brain and plasma of Sandhoff mice, and effect of the modified Hex B on cleavage of lyso-GM2 accumulated

**Figure 3:**
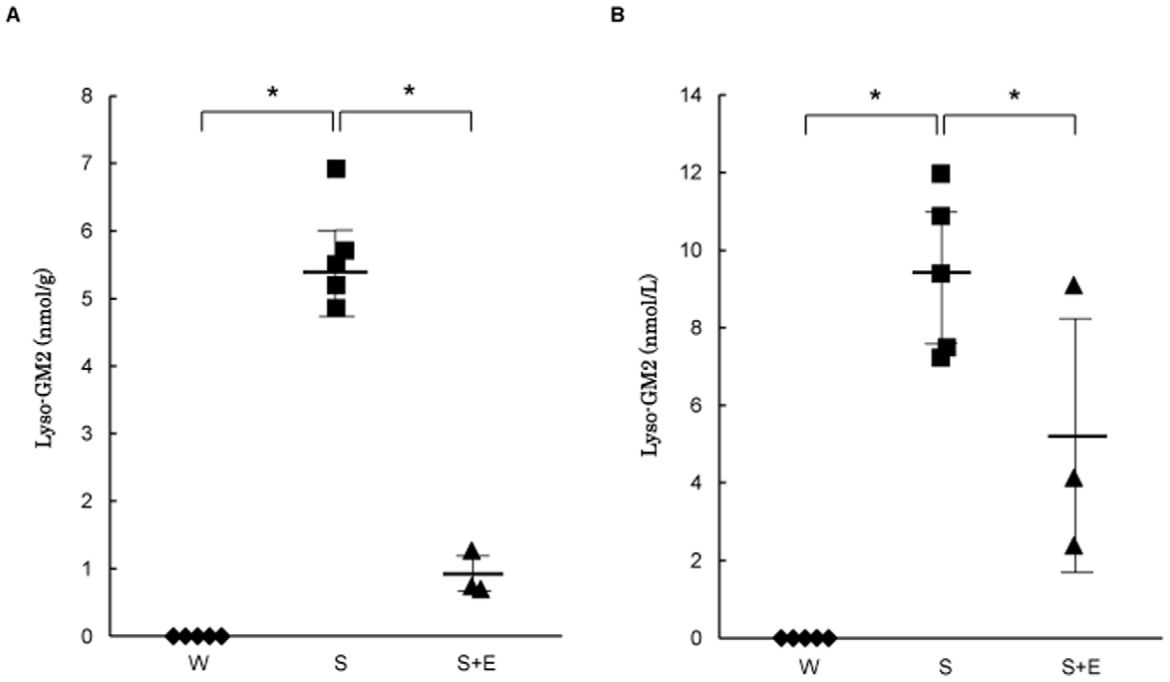
Lyso-GM2 levels in the brains and plasma of mice. Lyso-GM2 levels in brain tissues (**A**) and plasma (B). Wild-type mice (W,♦), untreated Sandhoff mice (S,▪), and Sandhoff mice treated with the modified Hex B (S+E,▴). Each line indicates the mean±SD. *P<0.05 (*t*-test).

Fig. 3 shows the lyso-GM2 levels in the brain and plasma of mice. Brain tissues from the wild-type mice exhibited lyso-GM2 levels below the detection level of 0.02 nmol/g. However, those in the Sandhoff mice were apparently increased to 5.6±0.8 nmol/g (mean±SD). The lyso-GM2 levels in the brain tissues from the Sandhoff mice treated with the modified Hex B decreased to 0.9±0.4 nmol/g (Fig. 3a) compared with those in the untreated Sandhoff mice.

Although the lyso-GM2 levels in the plasma of the wild-type mice were below the detection level of 1.0 nmol/L, those in the Sandhoff mice were apparently increased (9.4±2.1 nmol/L, mean±SD). The plasma lyso-GM2 levels in the Sandhoff mice treated with the modified Hex B were decreased (5.2±3.5 nmol/L, mean±SD), compared with those in the untreated Sandhoff mice except for one case (9.1 nmol/L). The results of the plasma lyso-GM2 analysis are summarized in Fig. 3b. The results are correlated with those in the case of brain lyso-GM2.

GM2 levels in the brain of Sandhoff mice and effect of the modified Hex B on cleavage of GM2 accumulated.

**Figure 4:**
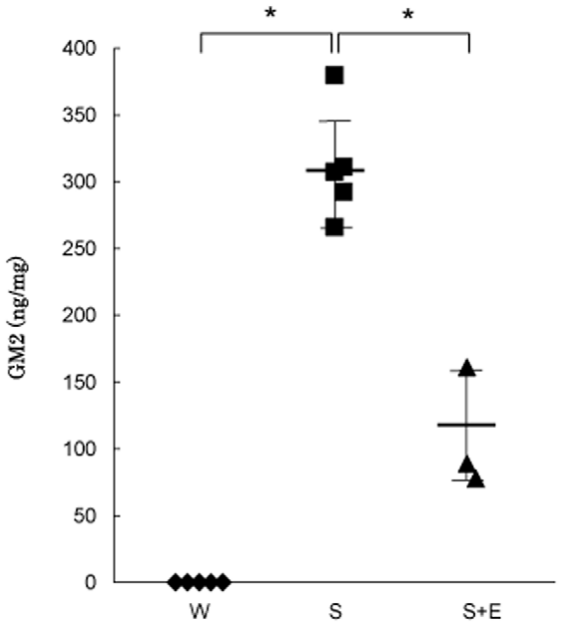
GM2 levels in the brains of mice. GM2 levels in brain tissues of wild-type mice (W,♦), untreated Sandhoff mice (S,▪), and Sandhoff mice treated with the modified Hex B (S+E,▴). Each line indicates the mean±SD. *P<0.05 (*t*-test).

Fig. 4 shows the GM2 levels in brain tissues from the wild-type mice, and the Sandhoff ones treated and not treated with the modified Hex B. Brain tissues from the wild-type mice exhibited GM2 levels below 10 ng/mg. On the other hand, the brain GM2 levels in the Sandhoff mice were apparently increased (312±42 ng/mg, mean±SD). The GM2 levels in the Sandhoff mice treated with the modified Hex B were decreased (109±45 ng/mg, mean±SD), compared with those in the untreated Sandhoff mice. The results were correlated with those in the case of brain lyso-GM2, although the effect of the modified Hex B on cleavage of the GM2 accumulated was lower than that in the case of lyso-GM2.

### Lyso-GM2 levels and Hex activities in human plasma

Table 1 summarizes the lyso-GM2 concentrations, and MUG- and MUGS-degrading activities in plasma samples from patients with Sandhoff disease, Tay-Sachs disease and GM2A deficiency, their parents, and controls. Plasma samples from the control subjects exhibited lyso-GM2 levels below 2.0 nmol/L. The plasma lyso-GM2 levels in the patients with Sandhoff disease and Tay-Sachs disease were apparently increased, i.e., an infantile Sandhoff case, 12.7 nmol/L, an adult Sandhoff one, 2.9 nmol/L, and an infantile Tay-Sachs one 32.7±5.1 nmol/L (means±SD, n = 5). On the other hand, the plasma lyso-GM2 level in the patient with GM2A deficiency was normal (< 2.0 nmol/L). The parents of Sandhoff disease and Tay-Sachs disease patients exhibited normal lyso-GM2 levels as expected.

**Table 1:** Plasma lyso-GM2 levels and hexosaminidase activities.

Disease	Case	Age	Sex	MUG-degrading activity (nmol/h/mL)	MUGS-degrading activity (nmol/h/mL)	Lyso-GM2 (nmol/L)	Reference
Sandhoff disease	Patient 1	1 y	M	17	5	12.7	[9]
	Patient 2	31 y	M	42	5	2.9	[10]
	Parent 1-1	unknown	M	309	34	<2.0	-
	Parent 1-2	unknown	F	357	41	<2.0	-
Tay-Sachs disease	Patient 3	1 y	F	552	2	39.9	-
	Patient 4	1 y	F	533	2	29.7	-
	Patient 5	1 y	M	382	2	26.4	-
	Patient 6	1 y	M	494	2	30.1	-
	Patient 7	2 y	M	281	1	32.6	-
	Parent 3-1	31 y	M	542	27	<2.0	-
	Parent 3-2	33 y	F	513	31	<2.0	
	Parent 4-1	unknown	M	347	30	<2.0	-
	Parent 4-2	unknown	F	478	26	<2.0	-
	Parent 5-1	31 y	F	1.12×10^3^	35	<2.0	-
GM2A deficiency	Patient 8	1 y	M	1.42×10^3^	81	<2.0	[11]
Control (n = 48)			M/F	709±302	58±14	<2.0	

Patient 1, and parents 1-1 and 1-2 are from the same family; patient 3, and parents 3-1 and 3-2 are from the same family; patient 4, and parents 4-1 and 4-2 are from the same family; and patient 5, and parent 5-1 are from the same family.

The Sandhoff patients exhibited decreases in both the MUG- and MUGS-degrading activities, which reflected the combined deficiency of Hex A and Hex B. The enzyme activities in the adult case were moderately higher than those in the infantile one. The decrease in enzyme activity was correlated with an increase in lyso-GM2. All of the infantile Tay-Sachs patients exhibited a decrease in MUGS-degrading activity, which reflected a specific deficiency of Hex A. The patients with GM2 activator deficiency exhibited normal enzyme activities for both Hex A and Hex B.

## Discussion

Although the details of the pathogenetic mechanisms leading to sphingolipidoses have not been clarified yet, it is likely that excessive accumulation of sphingolipids causes cellular dysfunction, induction of cytokines and a defect of autophagy. Another possible pathogenetic mechanism may be the formation of lysosphingolipids. Galactosylsphingosine and glucosylsphingosine are known to accumulate in neural cells of patients with Krabbe disease and Gaucher disease, respectively [15], [16]. Furthermore, it has been reported that not sphingolipids but lysosphingolipids induce apoptosis of mouse neuroblastoma Neuro2a cells [17], and apoptosis has actually been found in neural tissues of mouse models of GM2 gangliosidosis, and patients with Tay-Sachs disease and Sandhoff disease [18]. Lysosphingolipids are cytotoxic and may well be the pathogenetic agent for the neural degeneration in these diseases.

In GM2 gangliosidosis, lyso-GM2 was found in the brain tissues of patients with Tay-Sachs disease and Sandhoff disease as well as GM2 [19]–[22]. The origin of this lyso-GM2 is obscure, but an elevated lyso-GM2 level in the brain should be associated with the loss of Hex A activity and may be related to the pathophysiologies of these diseases.

Biomarkers of Tay-Sachs disease and Sandhoff disease useful for diagnosis and assessment of the effect of enzyme replacement therapy, which will be introduced clinical medicine in the near future, are now strongly required. So, we paid attention to lyso-GM2 and examined the lyso-GM2 level in brains of Sandhoff mice and the effect of the modified Hex B on cleavage of the lyso-GM2 accumulated. Our investigation revealed that the brain lyso-GM2 level in Sandhoff mice was increased and the intracerebroventricular injection of the enzyme apparently decreased it. The effect of the enzyme on cleavage of lyso-GM2 was more impressive compared with that in the case of GM2. As lyso-GM2 is more hydrophilic than GM2, which aggregates as a component of cellular membranes, the lyso-GM2 level would be more strongly influenced by the administration of the hydrophilic enzyme than that of GM2.

Then, we established an assay method for measuring plasma lyso-GM2 with HPLC, and determined the plasma lyso-GM2 levels in mice. The plasma lyso-GM2 levels in Sandhoff mice were apparently increased and they decreased on administration of the enzyme. The assay procedure is easy and authentic lyso-GM2, as a standard, which is necessary for quantifying plasma lyso-GM2, is commercially obtained, although it is difficult to obtain a proper authentic GM2 as a standard for measuring plasma GM2 levels by means of tandem mass spectrometry because GM2 purified from animal tissues usually has heterogeneous fatty acids.

Finally, we examined the lyso-GM2 levels in human plasma. The results were well correlated with those of the animal experiment, and it was revealed that the plasma level of lyso-GM2 was increased in patients with Tay-Sachs disease and Sandhoff disease. The increase in lyso-GM2 was associated with a decrease in Hex A activity. A patient with GM2A deficiency exhibited a normal level of lyso-GM2. As a patient with GM2A deficiency has a sufficient amount of Hex A, it may degrade hydrophilic lyso-GM2 without GM2A.

In conclusion, lyso-GM2 is expected to be a potential biomarker of Tay-Sachs disease and Sandhoff disease.
